# Shedding light on ethylene metabolism in higher plants

**DOI:** 10.3389/fpls.2014.00665

**Published:** 2014-12-01

**Authors:** Maria A. Rodrigues, Ricardo E. Bianchetti, Luciano Freschi

**Affiliations:** Laboratory of Plant Physiology, Institute of Biosciences, Department of Botany, University of São Paulo, São Paulo, Brazil

**Keywords:** ACC, ACO, ACS, ethylene, light signaling, photoreceptors, phytochrome

## Abstract

Ethylene metabolism in higher plants is regulated by a wide array of endogenous and environmental factors. During most physiological processes, ethylene levels are mainly determined by a strict control of the rate-limiting biosynthetic steps responsible for the production of 1-aminocyclopropane-1-carboxylic acid (ACC) and its subsequent conversion to ethylene. Responsible for these reactions, the key enzymes ACC synthase and ACC oxidase are encoded by multigene families formed by members that can be differentially regulated at the transcription and post-translational levels by specific developmental and environmental signals. Among the wide variety of environmental cues controlling plant ethylene production, light quality, duration, and intensity have consistently been demonstrated to influence the metabolism of this plant hormone in diverse plant tissues, organs, and species. Although still not completely elucidated, the mechanisms underlying the interaction between light signal transduction and ethylene evolution appears to involve a complex network that includes central transcription factors connecting multiple signaling pathways, which can be reciprocally modulated by ethylene itself, other phytohormones, and specific light wavelengths. Accumulating evidence has indicated particular photoreceptors as essential mediators in light-induced signaling cascades affecting ethylene levels. Therefore, this review specifically focuses on discussing the current knowledge of the potential molecular mechanisms implicated in the light-induced responses affecting ethylene metabolism during the regulation of developmental and metabolic plant responses. Besides presenting the state of the art in this research field, some overlooked mechanisms and future directions to elucidate the exact nature of the light–ethylene interplay in higher plants will also be compiled and discussed.

## INTRODUCTION

Light is one of the most influential and versatile environmental stimuli controlling plant life. It varies not only in quantity (fluence) but also in quality (wavelength), periodicity (photoperiod), and direction (unidirectional or diffuse). As photoautotroph and sessile organisms, most higher plants rely on sophisticated and plastic mechanisms to use light as both an energy source and abiotic signal to control decisive developmental adjustments. Not unexpectedly, during evolution plants have evolved a variety of photosensory systems that perceive the light environment and integrate this information into intrinsic developmental programs. Acting at the interface between the external and the internal plant environments, four main families of photoreceptors are responsible for the perception of light signals and their transduction through an array of gene expression modifications that will ultimately lead to adjustments in the plant growth and morphogenic patterns—a sequence of light-triggered processes collectively known as photomorphogenesis (Neff et al., 2000; Ma et al., 2001; Gyula et al., 2003; Franklin et al., 2005; Franklin and Quail, 2010).

During photomorphogenic responses the initial light cue is often translated into changes in the hormonal homeostasis in particular tissues or even throughout the entire plant. Hence, by modulating their hormonal status plants can rapidly adjust growth fitness to constantly changing environments (Li et al., 2004; Achard et al., 2006; Wolters and Jürgens, 2009). The early development of seedlings, which in nature is often skotomorphogenic, and the transition to photomorphogenic growth upon light exposure is one of the best-studied light-controlled processes in eudicotyledons, and it is tightly regulated by an intricate interplay between light signals and plant hormones. In fact, most of the current knowledge about light signaling pathways and ethylene biosynthesis, signal transduction, and response pathways has arisen from experimental approaches based on the etiolated seedling system. The simplicity of the light-induced responses in dark-grown seedlings made it ideal for quickly identifying photomorphogenic mutants in the model plant *Arabidopsis thaliana* (Liscum and Hangarter, 1993; Stepanova and Ecker, 2000; Li et al., 2004; Stepanova and Alonso, 2005; Chen and Chory, 2011; Boron and Vissenberg, 2014). In addition, the triple response phenotype of etiolated seedlings triggered by ethylene has also been proven as a simple and useful trait to screen for ethylene mutants in the same model plant (Bleecker et al., 1988; Guzman and Ecker, 1990; Lin et al., 2009).

Ethylene is an important growth regulator of numerous developmental aspects during plant life cycle (e.g., both vegetative and reproductive development, and responses to biotic and abiotic stresses), playing a key role in signaling pathways responsible for adaptive adjustments of plant’s fitness in a continuously fluctuating array of environmental signals. Moreover, ethylene biosynthesis is highly regulated by both developmental and external inputs, and stress-induced ethylene production can mediate multiple physiological and morphological responses involved in redirecting all required resources from standard growth to promote plant defense, resistance, resource forage, and/or “escape” mechanisms, including senescence, abscission, and plastic alterations in tissue/organ elongation and shoot–root ratios. However, the variable degree of plasticity observed in such ethylene-mediated responses seems to be dependent on the environmental challenge and species-specific features (Pierik et al., 2006, 2007; Lin et al., 2009; Wolters and Jürgens, 2009).

The development of photomorphogenic and hormonal mutant collections together with a considerable improvement in the experimental methods employed in molecular and genetics research over the last years have paved the way for elegant studies that have begun to shed light on the “black box” of how light signaling controls ethylene production to coordinate plant development. For example, it has been shown that light can modulate ethylene biosynthesis through particular photoreceptor-mediated pathways. Moreover, some transcription factors have been identified as potential integrators for light signaling and ethylene biosynthesis modulation. Furthermore, many regulators of ethylene biosynthesis at both transcriptional (Zhang et al., 2009; Wan et al., 2011; Xiao et al., 2013) and post-transcriptional levels (Wang et al., 2002, 2004; Chae et al., 2003; Liu and Zhang, 2004; Argueso et al., 2007; Joo et al., 2008; Prasad et al., 2010; Wan et al., 2011) have also been documented in the last decades. Besides controlling ethylene biosynthesis, light also influences ethylene signaling and, although not covered in this review, this is also an important aspect that has received increasing attention in the last decades.

Here we have compiled the major controlling mechanisms of both light signaling and ethylene biosynthesis and discussed the latest information on the potential signaling networks connecting both these pathways, with an emphasis on the emerging evidence of molecular mechanisms regulating the transcription and post-translational modifications of key participants in the ethylene biosynthetic pathway.

## LIGHT PERCEPTION AND SIGNALING MECHANISMS

Light perception and signal transduction are responsible for modulating many processes throughout the plant life cycle and in diverse environmental contexts. Among the different wavelengths detected by the plant photosensory system, red light (RL), blue light (BL), UV-A and UV-B radiation are particularly informative and their perception involves four main families of information-transducing photoreceptors: the RL-absorbing phytochromes, the UV-A/BL-absorbing cryptochromes and phototropins, and the recently identified UVR8 receptors, which essentially perceive UV-B radiation. These signaling molecules provide the plants with information concerning various aspects of the light environment, thereby playing a vital role in plant survival and optimal growth (Franklin et al., 2005; Franklin and Quail, 2010; Rizzini et al., 2011). Detailed signaling mechanisms triggered by photoreceptors have been the focus of excellent reviews (Quail, 2002; Gyula et al., 2003; Chory, 2010; Chen and Chory, 2011); therefore, only general information about this theme will be provided here.

### IDENTITY AND GENERAL FEATURES OF PLANT PHOTORECEPTORS

**FIGURE 1:**
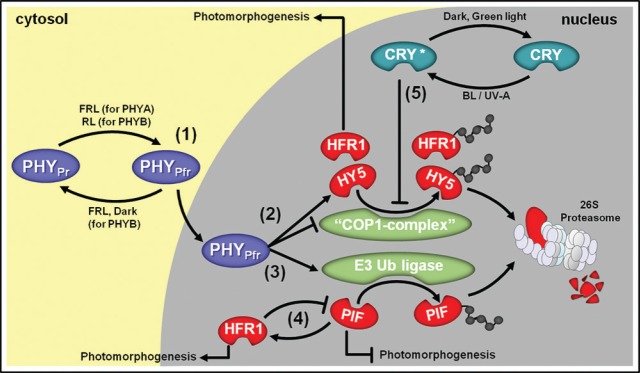
**Simplified overview of light signal transduction via phytochromes (PHY) and cryptochromes (CRY) and their main interacting proteins. (1)** Upon far-red light (FRL) or red light (RL) irradiation, cytosolic PHYA_Pr_ and PHYB_Pr_, respectively, are converted into their active Pfr forms, which migrate to the nucleus. **(2)** In the cell nucleus, PHY_Pfr_ represses CONSTITUTIVE PHOTOMORPHOGENIC 1 (COP1) E3-complex (“COP1 complex”), which is responsible for targeting positive components of the light signal transduction pathway such as LONG HYPOCOTYL 5 (HY5) and LONG HYPOCOTYL IN FAR-RED 1 (HFR1) to proteasomal degradation. The expression of genes coding for HY5 and HFR1 is also promoted by PHY_Pfr_ and these transcription factors are responsible for stimulating the expression of numerous photomorphogenesis-associated genes. **(3)** In parallel, PHY_Pfr_ activates an unidentified E3 Ubiquitin (Ub) ligase responsible for targeting light signal transduction repressor proteins such as PHYTOCHROME-INTERACTING FACTORS (PIFs) to degradation via 26S proteasome. **(4)** PIFs are also known to stimulate HFR1 and HFR1 blocks PIFs transcriptional activity. **(5)** Upon blue light (BL) or UV-A exposure, nuclear-localized CRY is converted into active CRY*, which also repress “COP1 complex” action.

Phytochromes are the RL and far-red light (FRL) photoreceptors that play essential roles during plant photomorphogenesis (Chen and Chory, 2011). They are part of a chromoprotein multigene family, which in *Arabidopsis* is divided into photodegradable (phytochrome A—PHYA) and photostable (phytochrome B, C, D, and E—PHYB, PHYC, PHYD, and PHYE) types. These photoreceptors are formed by the association of one globular apoprotein and one chromophore which confers the properties of activation in response to the RL spectrum and photoreversibility upon FRL exposure (Wagner et al., 2005). The perception of RL is mediated by the chromophore (Wagner et al., 2005), which is synthesized in plastids and combined with the phytochrome apoproteins in the cytosol. Under dark conditions, the chromophore is maintained in a stable-inactive conformation (PHY_Pr_) whereas the RL triggers the photoconversion of the inactive state PHY_Pr_ into the active form (PHY_Pfr_), resulting in the phytochrome translocation from the cytoplasm into the nucleus (Ulijasz et al., 2010; Song et al., 2011; Figure 1). Despite the relatively well-conserved structure, PHYA and PHYB have different response patterns in terms of light stimulation and action mechanisms. PHYA-mediated responses are mainly triggered by FRL and this phytochrome does not show photoreversibility (Quail, 2002). Once in the active form, PHYA_Pfr_ associates with the proteins FAR RED ELONGATED HYPOCOTYL (FHR) and FHR-LIKE (FHL) and transiently accumulates in the nucleus (Kircher et al., 2002; Pfeiffer et al., 2012). On the other hand, PHYB is primarily activated by RL, presents R/FR photoreversibility, and accumulates in the nucleus for longer periods than PHYA (Gil et al., 2000). Furthermore, distinct members of the PHY family can display a certain degree of organ specificity in response to RL (Tepperman et al., 2004).

The cryptochromes (CRY) are nuclear proteins that primarily respond to BL and UV-A radiation (Ahmad and Cashmore, 1997) and present an intricate interaction with phytochromes during the control of numerous photomorphogenic responses (Guo et al., 2001; Facella et al., 2008). In *Arabidopsis*, cryptochrome 1 (CRY1) is photostable and acts mainly at high light fluence (Yang et al., 2000; Herbel et al., 2013) whereas cryptochrome 2 (CRY2) is photodegradable and plays an important role at relatively lower light fluence (Lin et al., 1998; Guo et al., 2001; Gyula et al., 2003). CRY1/2 phosphorylation and subsequent molecular modifications are triggered soon after BL/UV-A exposure, facilitating the CRY interaction with other proteins (Kondoh et al., 2011). Such BL/UV-A-driven modifications in the CRY molecules can be reversed in complete darkness or upon exposure to green radiation (Sellaro et al., 2010).

In contrast, phototropins 1 and 2 are membrane-localized photoreceptors that undergo auto-phosphorylation in response to BL/UV-A radiation (Christie, 2007) and play an important role in many phototropic responses in plants (Kinoshita et al., 2001; Sakai et al., 2001). In the case of the UVR8 receptors, the perception of UV-B radiation occurs via tryptophan residues present at the protein homodimeric interface which leads to the monomerization and subsequent association of the UVR8 monomers with CONSTITUTIVE PHOTOMORPHOGENESIS 1 (COP1), which functions as an E3 ubiquitin ligase in multiple protein complexes and represents a master repressor during light signaling cascades. Such protein interaction will ultimately result in differential gene expression (Rizzini et al., 2011; Wu et al., 2012), and its potential outputs will be discussed later in the context of light signaling mechanisms controlling the transcription of genes involved in ethylene biosynthesis.

### HUB TRANSCRIPTION FACTORS UNDER PHYTOCHROME CONTROL

Downstream of the RL perception, an increasing number of transcription factors has been identified as central integrators of light and multiple internal signals to optimize plant development. A key mechanism by which PHYs regulate gene expression is by modulating the protein stability of target transcription factors in the nucleus. One of the most important PHY_Pfr_-mediated signaling pathways relies on the regulation of a multigene family of basic helix–loop–helix transcription factors known as PHYTOCHROME INTERACTION FACTOR (PIF). Accordingly, under inductive light conditions PHYA_Pfr_ and/or PHYB_Pfr_ migrates to the nucleus, where they can physically interact with PIF proteins and promote their ubiquitination and subsequent degradation via proteasome 26S (Figure 1). Under continuous darkness, PIF proteins inhibit the transcription of many genes associated with photomorphogenic responses; therefore, PHY activation and migration to the nucleus followed by its positive influence on PIF degradation represents a major point of transcriptional control of such genes under PIF-dependent inhibition (Ni et al., 1999; Khanna et al., 2004; Shen et al., 2007; Leivar et al., 2008; Lorrain et al., 2008; Chen and Chory, 2011). Besides playing a central role during the scoto-to-photomorphogenesis transition, PIFs are also involved in many developmental processes triggered by low R/FR conditions, such as shade avoidance responses, during which they control the transcription of genes coding for important proteins such as PIF3-LIKE 1 (PIL-1) and ARABIDOPSIS THALIANA HOMEOBOX PROTEIN 2 (ATHB2), and some components of the biosynthetic routes of plant hormones such as auxins, gibberellin, and ethylene (Martínez-Garcia et al., 2000; Leivar et al., 2009).

Active PHYA_Pfr_ and PHYB_Pfr_ are also known to increase the levels of LONG HYPOCOTYL 5 (HY5), which is another crucial transcription factor associated with light signaling and several photomorphogenic responses in plants. Hence, under continuous darkness HY5 undergoes very rapid destabilization mediated by COP1 (Gyula et al., 2003; Chory, 2010; Boron and Vissenberg, 2014). In fact, COP1 usually acts in association with other regulatory proteins (“COP1 complex”) promoting the ubiquitin-dependent degradation of both HY5 and PHYs, thereby representing an important element in the post-translational regulation of the PHY-dependent signaling cascades. In an intricate crosstalk with PHY, CRY also plays a fundamental role in the stabilization of HY5 since both CRY1 and CRY2 have been shown to increase the half-life of this protein by directly interacting with COP1 and promoting its destabilization or removal from the cell nucleus (Figure 1), which ultimately leads to an extended window of time for the stimulatory action of HY5 on the transcription of genes associated with photomorphogenic responses. Moreover, CRY also increases the half-life of PHYA by destabilizing the COP1 complex (Schwechheimer and Deng, 2000; Gyula et al., 2003; Chory, 2010; Fankhauser and Ulm, 2011; Boron and Vissenberg, 2014).

### INFLUENCE OF DIVERSE LIGHT REGIMES ON ETHYLENE EVOLUTION

Light influence on ethylene evolution rate has been consistently demonstrated in several plants and can either stimulate or inhibit ethylene production depending on the tissue, organ, species, plant developmental phase, and the nature of light signal (Abeles et al., 1992; Corbineau et al., 1995; Halliday and Fankhauser, 2003; Kurepin et al., 2010). For example, European production of lilies can be hampered by long periods of low light, which induce higher production of ethylene, causing the abscission of developing flower buds (Abeles et al., 1992). On the other hand, potato plants treated with continuous light for faster tuber development displayed up to 15-fold increase in ethylene production, while ethylene levels rapidly dropped when these plants were transferred back to the 12 h light/dark photoperiod (Wheeler and Tibbitts, 1986). Despite the physiological response, most light-triggered adjustments in ethylene production seem to be mainly under PHY and CRY control (Vandenbussche et al., 2003; Kurepin et al., 2010).

PHY_Pfr_-mediated signaling usually represses ethylene emission in several monocotyledons and eudicotyledons, as reported for etiolated seedlings of pea (*Pisum sativum*; Goeschl et al., 1967; Kang and Burg, 1972; Steed et al., 2004), light-grown seedlings of sorghum (*Sorghum bicolor*; Finlayson et al., 1998, 1999), coleoptiles and apical segments of etiolated rice (*Oryza sativa*) seedlings (Imaseki et al., 1971), light-grown leaves of both oat (*Avena sativa*; Corbineau et al., 1995) and wheat (*Triticum aestivum*; Jiao et al., 1987), and adult plants of both tobacco (*Nicotiana tabacum*; Pierik et al., 2004a) and *Arabidopsis thaliana* (Vandenbussche et al., 2003; Bours et al., 2014). Given its important role in RL perception and signaling, PHYB is the photoreceptor more closely associated with the negative regulation of ethylene levels in several plant models (Finlayson et al., 1998; Vandenbussche et al., 2003). In addition, the inhibitory effect of RL on ethylene emission depends on plant light exposure duration, radiation fluence (Imaseki et al., 1971; Pierik et al., 2004b; Kurepin et al., 2010), and some light-evoked adjustments on the plant circadian clock (Finlayson et al., 1998). However, in some plant systems PHYA also seems to play a prominent role in repressing ethylene production by direct action and/or via CRY1 repression, as reported for de-etiolating *phyAphyB* mutant pea seedlings (Foo et al., 2006).

## CENTRAL METABOLIC INTERMEDIATES AND ENZYMES IN ETHYLENE BIOSYNTHESIS

Ethylene is a relatively simple unsaturated two-carbon gas which can be produced by numerous non-biological chemical reactions catalyzed by heat, oxidation, light, or ionizing radiation. In fact, non-biological model systems were systematically employed during the initial search for metabolic components and steps involved in ethylene production (Yang and Hoffman, 1984; Abeles et al., 1992). Such approaches led to the unexpected discovery that ethylene could be chemically generated from the amino acid methionine and its derivatives (Yang et al., 1966). Following this breakthrough, significant advances were achieved in ethylene research with biological systems, providing the basis of knowledge regarding the ethylene biosynthetic pathway (Yang and Hoffman, 1984; Abeles et al., 1992; Kende, 1993).

Ethylene biosynthesis in higher plants is now well characterized by a relatively simple metabolic pathway, which is, however, coordinated with some other equally important synthetic pathways involved in the plant metabolism regulation (e.g., polyamines). The identification of the intermediate components in ethylene biosynthesis allowed further elucidation of the two committed reactions in this pathway, which comprise the rate-limiting enzymes 1-aminocyclopropane-1-carboxylic acid (ACC) synthase (ACS, EC 4.4.1.14) and ACC oxidase (ACO, EC 1.4.3; Yang and Hoffman, 1984; Kende, 1993; Zarembinski and Theologis, 1994; Argueso et al., 2007; McClellan and Chang, 2008; Harpaz-Saad et al., 2012). Both ACS and ACO are encoded by multigene families whose members have been well characterized in some plant species and they are recognized as major players in ethylene biosynthetic regulation. However, as we will discuss in more detail below, the regulatory mechanisms of ethylene biosynthesis usually converge on the modulation of ACS proteins (Fluhr and Mattoo, 1996; Wang et al., 2002).

### YANG CYCLE AND SYNTHETIC PATHWAYS ASSOCIATED WITH ETHYLENE PRODUCTION

**FIGURE 2:**
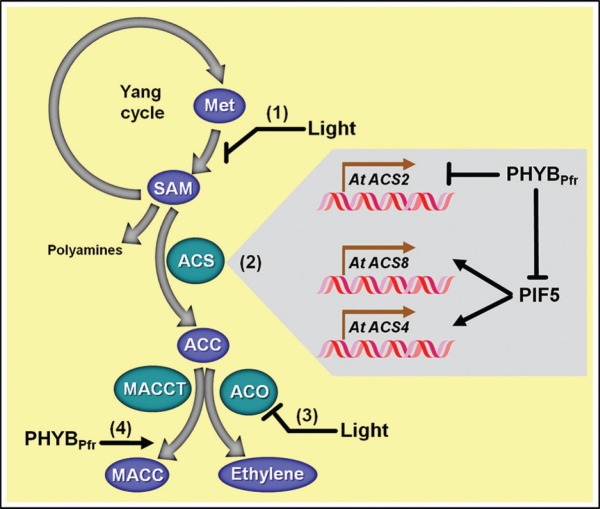
**Schematic representation of light-evoked impacts on ethylene biosynthetic pathway. (1)** Light irradiation negatively impacts the conversion of methionine (Met) to S-adenosyl methionine (SAM). **(2)** Light also affects the transcription of particular 1-aminocyclopropane 1-carboxylic acid (ACC) synthases (ACS), responsible for converting SAM into ACC. In *Arabidopsis*, whereas *ACS2* gene transcription is negatively controlled by PHYB_Pfr_, *ACS8/4* gene transcription is stimulated by PIF5, and, accordingly, PIF5 protein stability is negatively influenced by active phytochrome B (PHYB_Pfr_). **(3)** Light is also known to inhibit the transcription and activity of ACC oxidase (ACO), whose activity converts ACC into ethylene. **(4)** PHYB_Pfr_ also stimulates the conversion of ACC into the non-volatile ACC metabolite 1-malonyl aminocyclopropane-1-carboxylic acid (MACC) via MACC transferase (MACCT). Biosynthetic enzymes and metabolic substrates or products are represented with green and blue ovals, respectively. Light-dependent transcriptional control of particular ACS genes is highlighted by the gray area of the figure.

Ethylene biosynthesis *in planta* starts with the conversion of L-methionine to S-adenosyl methionine (AdoMet or SAM) in a reaction catalyzed by the enzyme L-methionine–S-adenosyltransferase (SAM synthetase, EC 2.5.1.6; Figure 2). The subsequent conversion of SAM to ACC is catalyzed by ACS and is generally considered the first committed and rate-limiting step in ethylene biosynthesis. The following crucial step in this pathway is catalyzed by ACO in a reaction that converts ACC to ethylene, CO_2_, HCN, and H_2_O (Adams and Yang, 1977, 1979; Lürssen et al., 1979; Yang and Hoffman, 1984; McKeon and Yang, 1987; Argueso et al., 2007; Lin et al., 2009). Behind this relatively linear sequence of few reactions hides a fundamental metabolic mechanism that prevents potential depletion of the methionine pools when a high rate of ethylene production is required. Such metabolic detail is significant because methionine, the biological precursor of ethylene, is a very scarce sulfur-containing amino acid and the amounts of sulfur available are usually limited in plants. In this sense, the methylthio-group from methionine needs to be recycled after SAM production. This is possible because the conversion of SAM to ACC by ACS also produces 5′-methylthioadenosine (MTA) as a by-product that retains the reduced methylthio-group and is readily recycled back to methionine through the Yang cycle. Therefore, the Yang cycle facilitates the occurrence of high rates of ethylene biosynthesis without influencing the steady-state levels of methionine pool (Yang and Hoffman, 1984; Miyazaki and Yang, 1987; Argueso et al., 2007; Zheng et al., 2013).

Besides being the direct precursor of ethylene, ACC is also suggested as a cell-signaling molecule *per se*. ACC is a soluble molecule that seems to be translocated throughout different plant organs, and its translocation within flowers has been suggested to play an important role in floral senescence (Abeles et al., 1992; Harpaz-Saad et al., 2012; Yoon and Kieber, 2013a). Hence, it appears logical that most tissues can actively regulate the endogenous ACC levels during plant development. This function is mainly controlled by ACS and ACO; however, the cellular pool of ACC can be also modulated by other metabolic reactions leading to its conjugation (Yang and Hoffman, 1984; McKeon and Yang, 1987; Martin and Saftner, 1995; Peiser and Yang, 1998; Staswick and Tiryaki, 2004; Kombrink, 2012). Accordingly, ACC can be converted into ACC derivates, such as jasmonic acid-ACC (JA-ACC), γ-glutamyl-ACC (GACC), and 1-(malonylamino) cyclopropane-1-carboxylate (MACC). Both GACC and JA-ACC comprise minor moieties in the pool of ACC derivates, whose potential biological functions still remain poorly characterized. In this sense, the physiological significance of such derivates might be underestimated (Staswick and Tiryaki, 2004; Kombrink, 2012). Conversely, MACC formation seems to be a metabolic sink that allows depletion of ACC levels under certain conditions. This reaction is catalyzed by ACC-N-malonyltransferase (MACCT; Figure 2), an enzyme that displays increased activity during late stages of tomato fruit ripening (Yang and Hoffman, 1984). Some hypotheses suggest MACC as an end metabolite derived from ACC that can be easily accumulated (Yang and Hoffman, 1984; McKeon and Yang, 1987), while others consider MACC as a means for temporary storage of ACC in a non-reactive form which could be hydrolyzed back to ACC when needed for ethylene production (Hoffman et al., 1982; Jiao et al., 1986; Hanley et al., 1989).

### LIGHT SIGNALING ON METABOLIC REGULATION OF THE ETHYLENE PRECURSOR LEVELS

One of the mechanisms involved in the PHY_Pfr_-dependent regulation of ethylene production in plants precisely relies on regulating the abundance of the immediate ethylene precursor ACC. Although still not fully characterized at the molecular level, the active form PHYB_Pfr_ has been shown to promote the rapid conjugation of ACC into MACC during the light-induced seedling de-etiolation of wheat, thereby decreasing the internal ACC pool available for ethylene formation (Jiao et al., 1987; Figure 2). Interestingly, these events occurred without any modification in the extractable MACCT activity, indicating that light might have exerted its inductive effects by a mechanism other than increasing the MACCT levels (Jiao et al., 1987). Such mechanism could represent some potential post-translational control of MACCT yet unknown.

## POST-TRANSLATIONAL MECHANISMS CONTROLLING ETHYLENE BIOSYNTHESIS

Both ACS and ACO family members are suggested to be at certain degree under post-translational regulation by proteasome-mediated degradation (Chen et al., 2005). However, particular attention has been devoted to unraveling the molecular structure and regulatory mechanisms of ACS proteins in *Arabidopsis*. For this reason, the following topics will mainly focus on discussing the central mechanisms involved in both transcriptional and post-translational control of ACS members in this plant model. Likewise, some evidence on ACS and ACO regulation in other species will be also comparatively discussed when opportune.

**FIGURE 3:**
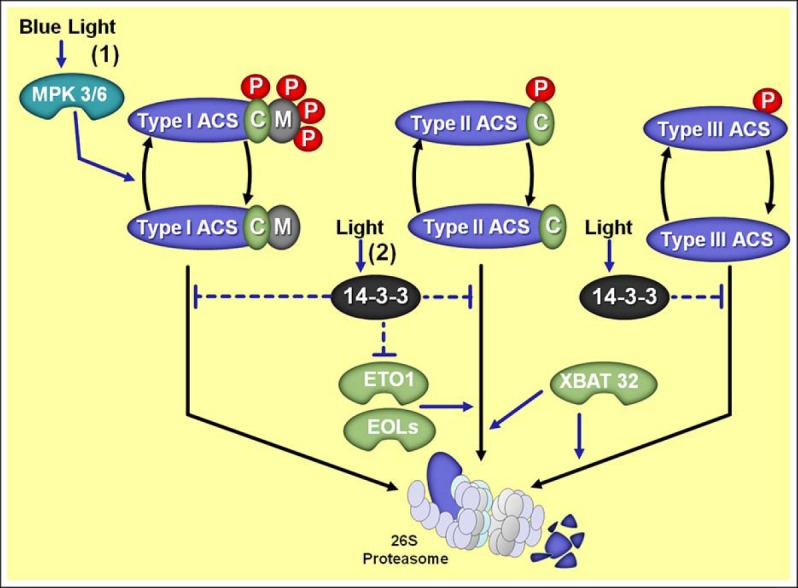
**Overview of the distinct regulatory mechanisms controlling stability of type I, II, and III 1-aminocyclopropane 1-carboxylic acid synthases (ACS) and their regulation by light. (1)** Blue light stimulates mitogen-activated protein kinases 3 and 6 (MPK 3/6) which in turn phosphorylate type I ACSs, thereby preserving ACSs from 26S proteasome-mediated degradation. **(2)** Light also stimulates 14-3-3 proteins which influence ACSs protein stabilization by both direct protein interaction and destabilization of ETHYLENE-OVERPRODUCER1 (ETO1) and ETO1/2-LIKE (EOL1/2) responsible for targeting type II ACS proteins for proteasomal degradation. Moreover, the RING-type E3 ligase XBAT32 affects both type II and III ACSs degradation. Protein phosphorylation is represented with red ovals and “P” letters. Phosphorylation sites targeted by calcium-dependent protein kinases (CDPK) are represented with green ovals and “C” letters. Phosphorylation sites targeted by MPK are represented with gray ovals and “M” letters. Note that type I ACS have both CDPK (“C”) and MPK (“M”) phosphorylation sites, type II ACS lacks only the MPK (“M”) phosphorylation site and type III ACS lacks both phosphorylation sites.

Briefly, ACS proteins belong to the α-superfamily of pyridoxal-5′-phosphate-dependent enzymes, which are represented in *Arabidopsis* by eight active members differentially regulated by several developmental and environmental signals. In addition to these active ACS enzymes, the *Arabidopsis* genome encodes an additional ACS (ACS1) that is catalytically inactive (Yamagami et al., 2003; Tsuchisaka and Theologis, 2004). Based primarily on the C-terminal sequence domains, ACS proteins are usually classified into three main groups which depict distinct regulatory features affecting the stability of the respective ACS proteins (Chae and Kieber, 2005; Yoshida et al., 2005). In *Arabidopsis*, type I ACS proteins (ACS2 and ACS6) have an extended C-terminus that contains phosphorylation sites (four conserved serine residues) targeted by mitogen-activated protein kinases (MPK) and calcium-dependent protein kinases (CDPK; Figure 3). Type II group includes isoforms (ACS4, ACS5, ACS8, and ACS9) with a shorter C-terminus that only contains a putative CDPK target site. Type III class is represented by one single member, ACS7, which has a very short C-terminal domain with no recognized phosphorylation sites (Tatsuki and Mori, 2001; Sebastià et al., 2004; Liu and Zhang, 2004; Chae and Kieber, 2005). Interestingly, this criterion of ACS classification has also been used for tomato ACS enzymes (Yoshida et al., 2005, 2006), indicating a general degree of structural conservation within ACS family. However, tomato encodes 10 LeACS isoenzymes with particular functional patterns (Yoshida et al., 2005, 2006).

### ACS PHOSPHORYLATION

Several experimental approaches have consistently suggested an important role for ACS phosphorylation and dephosphorylation during ethylene biosynthesis (Spanu et al., 1994; Tuomainen et al., 1997; Zhong and Burns, 2003), which regulates protein stability in an ACS isoform-dependent manner (Joo et al., 2008; Hahn and Harter, 2009; Figure 3). Genetic and biochemical studies have indicated CDPK and/or MPK as important molecules that cooperatively regulate stability of most ACS proteins and thus ethylene production, in response to developmental and environmental stimuli (Tatsuki and Mori, 2001; Liu and Zhang, 2004; Huang et al., 2013). As discussed previously, particular residues at the C-terminal domain of different isoforms of ACS represent putative target sites for phosphorylation, with MPK usually targeting three Ser residues that are distinct from the CDPK target site in the C-terminal extended region of a subset of ACS isoenzymes (Liu and Zhang, 2004; Sebastià et al., 2004; Joo et al., 2008; Huang et al., 2013).

Although very little information is available on CDPK signaling mechanisms during ACS regulation, it is widely accepted that the MPK3/6 module of MPK kinase (MKK) cascades plays relevant roles in the regulation of ethylene biosynthesis (Liu and Zhang, 2004; Joo et al., 2008; Xu et al., 2008; Hahn and Harter, 2009; Ju and Chang, 2012). Accordingly, the MPK3/6 module is mostly under control of MKK4, MKK5, and MKK9, constituting an important step in various signaling pathways involved in stress-induced responses in plants (Xu et al., 2008; Yoo et al., 2008; Beckers et al., 2009). For example, the stress-activated MKK4/5 signaling cascade can positively regulate ethylene biosynthesis by activating MPK6 (Liu and Zhang, 2004). In addition, ACS6 was initially identified as a potential substrate for MPK3/6 phosphorylation (Feilner et al., 2005) and further studies have confirmed that the MPK3/6 module is associated with enhanced stability of both type I ACS2 and ACS6 (Liu and Zhang, 2004; Joo et al., 2008; Figure 3). Interestingly, the autocatalytic ethylene production is often stress related and at least in *Arabidopsis* relies on post-translational regulation of type I ACSs by MPK3/6 cascade (Vandenbussche et al., 2012). Furthermore, the MKK9–MPK3/6 module is also suggested as a potential regulatory mechanism of ACS2/6 stabilization in some particular biological contexts (Popescu et al., 2009), supporting previous suggestions of the possible role of MKK9 and MPKs in ethylene biosynthesis control (Joo et al., 2008; Xu et al., 2008).

Therefore, ACS phosphorylation appears to be a major mechanism controlling the enzyme activity by increasing protein stability (Tsuchisaka and Theologis, 2004; Chae and Kieber, 2005; Argueso et al., 2007). Besides, tobacco plants under stressful conditions display a rapid increase in ethylene production mediated by an active NtMEK2 (MPKK)/SIPK (MPK) cascade that triggers a dramatic increase in ACS activity, which is followed by the transcriptional activation of a subgroup of *ACS* and *ACO* genes (Kim et al., 2003). As discussed in the following sections, recent data also suggest that phosphorylation might increase ACS affinity to directly interact with other proteins that influence ACS stability (Yoon and Kieber, 2013b).

### ACS DEGRADATION

As previously discussed, the ubiquitin–26S proteasome has been linked to diverse functions in plants, including hormone signaling, photomorphogenesis, and stress-triggered responses (Yi and Deng, 2005; Stone and Callis, 2007; Vierstra, 2009; Santner and Estelle, 2010). In fact, many aspects of ethylene biosynthesis are also highly regulated at the post-transcriptional level by degradation of proteins controlled by the ubiquitin–26S proteasome system. Genetic studies in *Arabidopsis* have begun to reveal a number of ethylene biosynthetic enzymes which are targeted for proteasomal degradation, including the type II ACS proteins and the type III ACS7 (Wang et al., 2004; Yoshida et al., 2005; Christians et al., 2009; Lyzenga et al., 2012). Although not fully understood, ample evidence indicates that protein phosphorylation preserves ACS proteins from 26S proteasome-mediated degradation (Figure 3), providing a direct and rapid mechanism to change ethylene production (Spanu et al., 1994; Kelley and Estelle, 2012).

Details on post-translational regulation of ACS proteins have been derived from analyses of the *ethylene-overproducing* (*eto1–3*) mutants of *Arabidopsis*. Accordingly, dark-grown *eto* seedlings display the triple-response phenotype even in the absence of exogenous ethylene application and produces 10- to 40-fold more ethylene in the dark than the wild-type seedlings (Guzman and Ecker, 1990; Woeste et al., 1999; Chae et al., 2003; Wang et al., 2004). The increased ethylene biosynthesis in *eto1* and *eto2* mutations results from two different modifications at the C-terminal region of ACS5 isoform, while *eto3* mutation is the result of a single amino acid change at the C-terminal region of ACS9 (Chae et al., 2003; Chae and Kieber, 2005). Such mutations were identified to cause disruptions within the TOE (for target of ETO1) domain of these ACS, which is recognized as a target site for ubiquitin–26S proteasome degradation. In fact, *ETHYLENE-OVERPRODUCER1 (ETO1)/ETO1/2-LIKE* (*EOL1/2*) genes code for CULLIN-3 E3 ubiquitin ligases which recognize and directly interact with the TOE domain of type II ACS proteins, targeting them for rapid degradation via 26S proteasome (Vogel et al., 1998; Chae et al., 2003; Wang et al., 2004; Yoshida et al., 2005, 2006; Christians et al., 2009; Figure 3). Additionally, the stability of both ACS7 (type III) and ACS4 (type II) was recently found to be turned over in a 26S proteasome-dependent manner through the participation of a RING-type E3 ligase named XBAT32 (Lyzenga et al., 2012), which also negatively modulates the abundance of ACS proteins and ethylene biosynthesis (Prasad and Stone, 2010; Prasad et al., 2010; Lyzenga et al., 2012). As the type III ACS7 lacks a C-terminal extension with the TOE sequence (Lyzenga et al., 2012), XBAT32 seems to represent a still unknown TOE-independent mechanism controlling ACS stability.

Adding an extra level of complexity in the ubiquitin–26S proteasome-dependent ACS stability control, recent findings revealed that ACS stability is also affected by ACS interaction with 14-3-3 proteins (Yoon and Kieber, 2013c; Figure 3). In this sense, 14-3-3 seems to interact with multiple isoforms from all three classes of ACS proteins, and this interaction increases the stability of the ACS proteins (Yoon and Kieber, 2013b). The molecular mechanism behind such regulation appears to involve the direct interaction of 14-3-3 with ACS proteins by decreasing their degradation by a still unknown ETO1/EOL-independent mechanism. These findings support the previous hypothesis that there is at least one further system acting to degrade type II ACS proteins in addition to the ETO1/EOLs, and the 14-3-3 proteins seem to antagonize this second degradation pathway (Lyzenga et al., 2012; Yoon and Kieber, 2013c). However, 14-3-3 proteins also seem to facilitate increased ACS stability by their interaction with the ETO1/EOLs E3 ligases which down-regulates their stability, thus increasing their degradation in an ubiquitin/proteasome-dependent manner and, consequently, decreasing the abundance of the ubiquitin ligases that target a subset of ACS proteins for degradation (Yoon and Kieber, 2013b).

In rice, the type II OsACS1 can interact with 14-3-3 proteins and the C-terminal domain of this enzyme was presumably phosphorylated by OsCDPK on a 14-3-3 recognition motif, suggesting that the phosphorylated OsACS1 may interact with rice 14-3-3 proteins, thus preventing ETO1 from binding to OsACS1 and induce this enzyme degradation (Yao et al., 2007). Therefore, such a regulatory mechanism might explain why after CDPK-dependent phosphorylation ACS proteins display increased protein stability (Yao et al., 2007; Yoon and Kieber, 2013c). Moreover, some evidence has indicated that 14-3-3 proteins interacted with ACO2 and ACO4 proteins in a yeast two-hybrid system, thus indicating that 14-3-3 proteins may regulate the ethylene biosynthesis pathway by modulating both ACS and ACO proteins (Huang et al., 2013).

### LIGHT SIGNALING AND THE POST-TRANSLATIONAL CONTROL OF ACS AND ACO PROTEINS

Recently, Yoon and Kieber (2013c) reported that etiolated seedlings of *Arabidopsis* submitted to light treatment present a rapid (within 2 h) increase in the levels ACS5 proteins without corresponding changes in *ACS5* transcripts, suggesting that light stimuli act by increasing ACS5 stability in this plant system. Accordingly, light treatment triggers the opposite effect on EOL2 protein levels, which exhibit a significant and rapid reduction as soon as 2 h after light exposure. These authors have suggested that the mechanism by which 14-3-3 proteins control ACS stability could also be regulated by light stimuli; however, such hypothetical connection remains uncertain. Furthermore, some evidence indicates that the particular isoform SbACO2 from sorghum might show a light-regulated post-transcriptional regulation throughout the diurnal cycle. This suggestion was based on the observation that diurnal fluctuations in SbACO2 transcript abundance were translated into diel changes in enzymatic activity under unshaded environment, but not under simulated high-shade conditions (Finlayson et al., 1999). Additionally, recent findings have shown significant connections between MPK and light signaling pathways in plants which indicate the MKK3–MPK6 cascade being actively regulated by BL signaling at several levels (Sethi et al., 2014). Therefore, it is tempting to speculate that MKK3–MPK6-dependent cascades might possibly be implicated in the interplay between light signaling and ACS post-translational control (Figure 3).

## TRANSCRIPTIONAL MECHANISMS CONTROLLING ETHYLENE BIOSYNTHESIS

Transcriptional regulation of *ACS* and *ACO* genes is one of the pivotal mechanisms controlling ethylene biosynthesis (Wan et al., 2011). As previously discussed, although regulatory mechanisms affecting ACS activity are generally considered the crucial regulatory point for ethylene production, increasing evidence has indicated that modulation of *ACO* expression can also represent a significant point of control for ethylene production under particular circumstances (Rudus et al., 2013; Xiao et al., 2013). Accordingly, a number of studies have reported that both ACS and ACO family members can be differentially expressed in diverse plant organ/tissues, distinct developmental phases, and in response to different environmental stimuli (Liang et al., 1992; Kende, 1993; Zarembinski and Theologis, 1994; Vahala et al., 1998; Wang et al., 2002; Argueso et al., 2007). Differences in the expression levels of each *ACS* and *ACO* genes might be an important means for adjusting differential ethylene production within a particular organ or plant tissue. For instance, the *ACS* multigene family of *Arabidopsis* has a prominent member-specific spatial regulation, suggesting a tissue-specific diversity of ethylene production (Tsuchisaka and Theologis, 2004). Likewise, members of the *ACO* gene family of tomato (*Solanum lycopersicum*) are also differentially regulated at the transcriptional level during climacteric fruit ripening, indicating distinct roles played by each *ACO* homolog during different tomato fruit developmental stages (Rudus et al., 2013; Xiao et al., 2013).

Additionally, both *ACS* and *ACO* expression can be directly and/or indirectly controlled by several signaling connections with other plant hormones such as auxin, brassinosteroid, and gibberellin (Guzman and Ecker, 1990; Joo et al., 2006). In this sense, auxin has been widely recognized as one of the most significant hormones controlling ethylene biosynthesis through transcriptional regulation of several *ACS* genes (Abel et al., 1995; Tsuchisaka and Theologis, 2004; Stepanova et al., 2007). In *Arabidopsis*, with the exception of *ACS1*/7/9 genes, the transcription of all other *ACS* members coding for functional enzymes are induced by auxin (Yamagami et al., 2003). In fact, the auxin–ethylene crosstalk often occurs via reciprocal regulation at biosynthetic level (Stepanova et al., 2007) with auxin apparently acting mostly via up-regulation of *ACS4* transcription in *Arabidopsis* (Liang et al., 1992; Abel et al., 1995; Zhu and Guo, 2008). On the other hand, some data on the crosstalk between auxin and ethylene during transcriptional regulation of two *ACO* genes in rice (*OsACO2*/3) have indicated that auxin effects on ethylene biosynthesis are highly dependent of the ethylene *status* itself. For example, *OsACO3* is induced by ethylene, but not in the presence of auxin, whereas *OsACO2* is induced by auxin, but in a reduced extent when in the presence of ethylene (Chae et al., 2000). Hence, auxin induction of *ACO* transcripts is generally considered an indirect effect of auxin-stimulated ethylene production resulting from an increase in ACS activity (Peck and Kende, 1995). Hence, direct regulation of *ACO* transcription seems to be mainly induced by ethylene itself by an autocatalytic process that will be discussed in the next sections of this review.

### ETHYLENE CONTROLLING ITS OWN BIOSYNTHESIS VIA ACS AND ACO TRANSCRIPTION

As mentioned previously, ethylene can modulate its own metabolism (Tsuchisaka et al., 2009) and this process usually occurs in an extremely flexible, developmentally and environmentally sensitive manner. Several studies have been reported on either positive or negative signaling feedback loops wherein ethylene regulates its own production (Sisler et al., 1985; Nakajima et al., 1990; Rottmann et al., 1991; Van Der Straeten et al., 1992; Woodson et al., 1992; Lincoln et al., 1993; Rodrigues-Pousada et al., 1999; Wang et al., 2002; Zhong and Burns, 2003). Therefore, depending on the plant tissue and the developmental/environmental context, ethylene can either restrain (auto-inhibition or system 1) or promote (autocatalysis or system 2) its own biosynthesis. Moreover, it has also been suggested that particular members of *ACS* gene family can play specific roles in these two systems of ethylene production in plants (Yang and Hoffman, 1984; Liu et al., 1985; Nakatsuka et al., 1998; Vandenbussche et al., 2012).

Negative feedback is particularly active during vegetative growth under non-stressful conditions and non-climacteric fruit development while autocatalytic ethylene production is usually related to stressful conditions, senescence and climacteric fruit ripening (McMurchie et al., 1972; Nakatsuka et al., 1998; Vandenbussche et al., 2012). During tomato fruit ripening different *ACS* and *ACO* genes are induced at particular stages of ripening. For example, *LeACS2* and *LeACS4* are mainly associated with autocatalytic ethylene production while *LeACS6* is mostly related to the auto-inhibitory system responsible for the maintenance of basal ethylene production (Nakatsuka et al., 1998; Barry et al., 2000; Cara and Giovannoni, 2008).

In *Arabidopsis*, the auto-inhibitory system also operates through the tightly controlled activities of several ACS proteins, which generate the basal ethylene levels in a coordinated manner (Yoshii and Imaseki, 1982; Tsuchisaka et al., 2009). The transcription of *ACS6* gene in this same species is generally associated with stress-induced conditions, in which ethylene autocatalysis plays an important role (Tsuchisaka et al., 2009; Vandenbussche et al., 2012). Furthermore, at least two *ACO* genes are ethylene inducible in *Arabidopsis*, suggesting that a feedback mechanism is in place to ensure that there will be no limitations to ethylene production once the precursor ACC is produced (Alonso et al., 2003; Zhong and Burns, 2003; Chen et al., 2005). Interestingly, the autocatalytic ethylene production in *Arabidopsis* is usually mediated by MPK cascade modules associated with stress-triggered signaling pathways. For example, besides post-translationally controlling ACS protein stability, the MPK3/6 module plays an important role in the ethylene autocatalytic pathway by inducing the transcription of the WRKY33 transcription factor which, in turn, directly interacts with *ACS2/6* promoters (Smékalová et al., 2014). In addition, besides triggering *ACS2*/6 transcription up-regulation, the MKK9-MPK3/6 module also positively regulates the transcript abundance of several *ERF* (*ETHYLENE RESPONSE FACTORS*) genes (Xu et al., 2008; Yoo et al., 2008).

*ERF* or *EREBP* (*ETHYLENE RESPONSE ELEMENT-BINDING PROTEIN*) genes represent one of the largest families of transcription factors in the plant kingdom which regulates a diverse array of processes (including ethylene production) in response to multiple developmental and environmental cues (Smékalová et al., 2014). These plant-specific transcription factors function as *trans*-acting regulators at the last step of ethylene signaling pathway, and usually exhibit highly conserved motifs that includes AP2 (APETALA2)/ERF DNA-binding domain, RAV (related to ABI3/VP1), and DREB (dehydration-responsive element binding protein). These ERF domains can specifically interact with *cis*-elements in promoters of the ethylene-responsive genes, such as GCC box and/or dehydration-responsive elements/C-repeat (DRE/CRT) motifs, thus regulating the expression of different downstream genes (Ohme-Takagi and Shinshi, 1995; Park et al., 2001; Huang et al., 2004; Lin et al., 2009; Zhang et al., 2009; Kohli et al., 2013). In addition, some ERFs can induce the transcription of additional transcription factors, inducing a transcription-factor cascade that might facilitate the signal amplification and fine-tuning of signal output (Chen et al., 2005).

Several studies have already shown that ERFs participate in a feedback regulation of ethylene production by modulating the expression of ethylene biosynthesis genes during fruit ripening (Zhang et al., 2009; Sharma et al., 2010; Lee et al., 2012; Pirrello et al., 2012). For example, ectopic expression of the *ERF.B3-SRDX* dominant repressor in tomato caused reduction in ethylene biosynthesis associated with the down-regulation of *ACS* and *ACO* genes, which presented the GCC box and DRE/CRT motifs at their promoters (Liu et al., 2013; Pirrello et al., 2012). In addition, LeERF2/TERF2 activates ethylene biosynthesis by promoting the expression of *ACS* genes in tobacco and of both *ACS* and *ACO* genes in tomato (Zhang et al., 2009). On the other hand, the ERF repressor SlAP2a opposes tomato ripening by suppressing the expression of *ACS2/4* and *ACO1* genes, then causing down-regulation of ethylene biosynthesis (Chung et al., 2010). Interestingly, distinct *MaERFs* of banana (*Musa acuminata*) can be differentially expressed in a tissue-dependent manner during fruit ripening while different *MaERFs* appeared to regulate the transcription of particular *ACS* and/or *ACO* in either positive (MaERF9-induced activation of *MaACS1*) or negative (MaERF11-induced repression of *MaACS1* and *MaACO1*) way (Xiao et al., 2013). Therefore, although the important role of multiple ERFs in the regulation of fruit ripening has been consistently described by several studies, little information is available about the signaling pathways involved in ERF regulation and the additional mechanisms modulating their target genes.

Furthermore, ERF proteins also play important roles in plant response to a variety of stress-related stimuli that involves modulation of ethylene synthesis (Xu et al., 2006; Wang et al., 2008; Hattori et al., 2009; Yaish et al., 2010). For instance, the ERF genes *SUB1A* and *OsDERF1* of rice are, respectively, positive and negative regulators in drought response. Upon drought stress, the expression of both these ERFs is modulated, affecting in turn the ethylene production via alterations in the expression of ethylene biosynthesis genes (Xu et al., 2006; Fukao et al., 2011; Wan et al., 2011). Consequently, it has become clear that ERF transcription factors are the primary targets mediating stress-induced responses involving changes in ethylene signaling and/or biosynthesis. However, very little is known about the signaling mechanisms controlling ERF proteins in ethylene biosynthesis at the transcriptional level (Zhang et al., 2009), which highlights the great demand to identify transcription factors and other signaling elements involved in ethylene biosynthesis adjustments. The potential regulatory role of these transcription factors during the light-induced pathway leading to the regulation of *ACS* and/or *ACO* transcription will be discussed below.

### LIGHT SIGNALING AND THE TRANSCRIPTIONAL CONTROL OF ACS AND ACO ENZYMES

PHYB is well known to module ethylene emission by repressing the transcription of genes encoding particular ACS isoenzymes (Figure 2). Illustrating this regulatory mechanism, mutations in *phyA* and *phyB* provide, respectively, increases on transcription of *ACS1* in pea (Foo et al., 2006) and *ACS2* in *Arabidopsis* (Bours et al., 2014). In fact, the genome-expression profiling of *Arabidopsis* revealed the light-regulated suppression of *ACS*, *ACO*, and *SAM synthase* transcripts (Ma et al., 2001). Accordingly, in this plant species the RL-evoked inhibition of ethylene emission not only depends on the repression of *ACS2* gene transcription, but is also strongly associated with the PHYB-dependent degradation of PIF proteins, such as PIF4 and particularly PIF5. Interestingly, PIF1 and PIF3 biochemically interact with both PHYA and PHYB, whereas PIF4 and PIF5 are only degraded when associated with active PHYB and this PHYB-mediated degradation of PIF4 and PIF5 is considered to be an important mechanism responsible for controlling ethylene production in plant responses triggered by either darkness or low R/FR conditions, such as skotomorphogenic growth and shade avoidance responses (Lorrain et al., 2008; Shin et al., 2009).

As previously described, PIF5 protein is constitutively localized in the nucleus, where it exerts a repressive influence on the transcription of genes associated with photomorphogenic responses and presents a positive impact on genes associated with low R/FR responses, such as *ACS4/8* (Figure 2). In agreement, *Arabidopsis* plants over-expressing *PIF5* exhibited up to 300-fold increases in *ACS4* transcripts even in the presence of light (Khanna et al., 2007). Although less prominent than observed for the *ACS4* expression, PIF5 also positively influences *ACS8* expression, which is the most abundantly expressed *ACS* gene in *Arabidopsis* and is the *ACS* gene most strongly controlled by endogenous ethylene and circadian clock (Yamagami et al., 2003; Thain et al., 2004). Due to such prominent impact on ethylene production, the *PIF5* over-expression in *Arabidopsis* resulted in the triple response phenomenon characteristically triggered by excessive ethylene production. Moreover, PIF5 is also known to destabilize PHYB by promoting its degradation via proteasome 26S, and this PIF5-triggered degradation of PHYB might accentuate even more the ethylene production in conditions of high PIF5 protein abundance (Khanna et al., 2007).

Furthermore, it has become clear that an integrated, multi-hormonal network is a common signaling mechanism involved in ethylene biosynthesis control (Chae et al., 2000). For example, besides acting as repressors of gibberellins (GA) responses, DELLA proteins can also physically interact with PIF proteins, thereby inhibiting PIF responses via a PHY-independent mechanism. Such a DELLA-dependent PIF suppression mainly takes place under complete darkness, when both these proteins are at abundant levels. Thus, the influence of GA on the transcriptional regulation of some ethylene-related genes, such as *ACS8*, seems to be associated with the DELLA destabilization and consequent repression of PIF degradation. Another overlap between ethylene and GA consists of the fact that active PHYB_Pfr_ represses active GA biosynthesis, which in turn decreases ethylene emission via DELLA-dependent and independent pathways (Keller et al., 2011). Accordingly, low R/FR conditions promote DELLA degradation, leading to increased PIF-related responses (de Lucas et al., 2008) which include the stimulation of ethylene biosynthesis. Moreover, light responses triggered by low fluence radiation, such as shade avoidance, are well known to involve both auxins and ethylene, during which an intensive crosstalk between these phytohormones and PHYB is suggested (Vandenbussche et al., 2003; Millenaar et al., 2009; Pierik et al., 2009). For example, *Arabidopsis* mutants exhibiting low auxin sensitivity, such as *auxin resistant 2* (*axr2*) and (*axr1–3*), usually present phenotypical similarities to wild-type plants maintained under complete darkness or low R/FR radiation. Treatment of these mutants with ethylene has been shown to revert this phenotype, suggesting that low R/FR leads to increased auxin levels, and these elevated auxin levels are responsible for stimulating ethylene production under these conditions. Additionally, auxin and low R/FR apparently share the same signaling pathways to increase *ACS6/8* transcript abundance (Vandenbussche et al., 2003). However, some contrasting results obtained with low R/FR-treated *Arabidopsis* plants (Millenaar et al., 2009; Pierik et al., 2009) have indicated that signaling interactions among PHYs, auxins, and ethylene during low-light induced responses can be highly plastic and dependent on specific experimental conditions (i.e., plant material obtained by distinct growth methods, different periods of exposure to the light treatment).

Adding even more complexity to the crosstalk between light signaling and regulation of ethylene biosynthesis in plants, it is currently known that LONG HYPOCOTYL IN FAR-RED1 (HFR1) is particularly relevant during low F/FR responses. Accordingly, HFR1 is a nuclear protein structurally similar to PIF3 (Fairchild et al., 2000), which is phosphorylated by COP1 and usually marked for degradation under complete darkness (Figure 1). However, in the presence of light, especially under BL- or FRL-rich radiation, this protein remains much more stable due to the presence of active PHYA and CRYs. Some FRL-induced responses that are associated with modifications in ethylene emission (e.g., repression of *ACS8* transcription) are apparently influenced by the biochemical interaction between PHYA-HFR1 or via the heterodimerization of HFR1 with PIF3 (Fairchild et al., 2000). However, it is also possible that such FRL-induced responses associated with modifications in ethylene evolution occurs through a more indirect pathway involving the HFR1-induced repression of genes and/or enzymes of other plant hormone biosynthetic pathways, such as GA and auxin (Sessa et al., 2005). Recent work has also suggested that PIFs and COP1 complexes synergistically repress photomorphogenesis in the dark, indicating that PIF proteins might inhibit HY5 by direct and indirect mechanisms (Xu et al., 2014). Furthermore, COP1 was reported as capable of physically interact with PIF3-LIKE1 (PIL1) and promote PIL1 degradation via the 26S proteasome, whereas PHYB physically interacts with PIL1 and enhances PIL1 protein accumulation upon RL irradiation, possibly via suppressing the COP1–PIL1 association (Jang et al., 2010; Luo et al., 2014).

Since PIF and COP1/HY5-mediated pathways are generally considered the two main light signaling branches downstream of the photoreceptors (Figure 1), it is likely that these components participate as central integrators between signaling pathways mediated by light and hormones cues (Yamamoto et al., 1998; Lau and Deng, 2010). In fact, some studies have consistently indicated PIF and COP/HY5 as hub steps of convergence and integration of signaling information mediated by both light and hormones. For example, such a PIF/COP/HY5-mediated network appears to control the opposite effects induced by ethylene on hypocotyl elongation of etiolated *Arabidopsis* seedlings when grown under darkness (elongation promotion) or light exposure (elongation repression; Feng et al., 2008; Liang et al., 2012). Interestingly, both these signaling steps are located downstream of ETHYLENE INSENSITIVE 3 (EIN3) during ethylene-regulated hypocotyl development in dark-grown seedlings (Ang and Deng, 1994; An et al., 2010). The transcription factors EIN3 and EIN3-LIKE 1 (EIL1) are intermediates in the ethylene signaling transduction responsible for the subsequent activation of *ERF* genes during multiple physiological processes (An et al., 2010; Liang et al., 2012). Moreover, COP1 appears to positively regulate the accumulation of EIN3 protein through a yet non-identified regulatory mechanism (Zhong et al., 2009) while COP1 affects the transcription of EIN3 downstream genes such as *ERF1* (Solano et al., 1998; Liang et al., 2012).

## CONCLUSION

Light has been acknowledged for some time as an important environmental cue capable of modulating ethylene biosynthesis in higher plants. While substantial progress has been made in identifying the metabolic components and enzymatic steps involved in ethylene biosynthetic route, our current understanding of how ethylene production is controlled by light signaling pathways is still very limited. Over the past decades, the identification of several mutants affected in particular aspects of ethylene production and/or photomorphogenic responses has facilitated significant advances in both research fields. As a result, some important breakthroughs on mechanistically explaining how light perception and signal transduction can modulate ethylene biosynthesis have been recently achieved, particularly in terms of the light-evoked changes in transcriptional and post-translational regulation of ACS and ACO enzymes.

Besides the direct effects of light-associated proteins on particular ethylene biosynthetic elements, we might also remain open-minded to conceive relatively more complex interconnection nodes, in which other plant hormones act in between the light perception and the actual modification of ethylene biosynthetic steps. Exciting new models of light–ethylene interaction networks might probably emerge when the combinatory influence of distinct photoreceptors (e.g., phytochromes and cryptochromes) or different environmental stimuli (e.g., light and biotic or abiotic stresses) started to be considered. Moreover, our knowledge about the mechanistic interplays between light and ethylene production might become even more complex as the research currently performed mainly in few plant models (e.g., *Arabidopsis*, tomato, pea) is extended to a wider range of species. Given the profuse influence of both light and ethylene on plant development, growth, and metabolism, studying the interplay between these stimuli is unarguably a promising research field for years to come.

### Conflict of Interest Statement

The authors declare that the research was conducted in the absence of any commercial or financial relationships that could be construed as a potential conflict of interest.
